# Measuring binding effects in event-based episodic representations

**DOI:** 10.3758/s13428-021-01769-1

**Published:** 2022-05-09

**Authors:** Marcel R. Schreiner, Thorsten Meiser

**Affiliations:** Department of Psychology, School of Social Sciences, University of Mannheim, L13, 15, 68161 Mannheim, Germany

**Keywords:** Statistical modeling, Episodic memory, Binding, Item response theory

## Abstract

Remembering an experienced event in a coherent manner requires the binding of the event’s constituent elements. Such binding effects manifest as a stochastic dependency of the retrieval of event elements. Several approaches for modeling these dependencies have been proposed. We compare the contingency-based approach by Horner & Burgess (*Journal of Experimental Psychology: General, 142*(4), 1370–1383, 2013), related approaches using Yule’s Q (Yule, *Journal of the Royal Statistical Society, 75*(6), 579–652, 1912) or an adjusted Yule’s Q (c.f. Horner & Burgess, *Current Biology, 24*(9), 988–992, 2014), an approach based on item response theory (IRT, Schreiner et al., in press), and a nonparametric variant of the IRT-based approach. We present evidence from a simulation study comparing the five approaches regarding their empirical detection rates and susceptibility to different levels of memory performance, and from an empirical application. We found the IRT-based approach and its nonparametric variant to yield the highest power for detecting dependencies or differences in dependency between conditions. However, the nonparametric variant yielded increasing Type I error rates with increasing dependency in the data when testing for differences in dependency. We found the approaches based on Yule’s Q to yield biased estimates and to be strongly affected by memory performance. The other measures were unbiased given no dependency or differences in dependency but were also affected by memory performance if there was dependency in the data or if there were differences in dependency, but to a smaller extent. The results suggest that the IRT-based approach is best suited for measuring binding effects. Further considerations when deciding for a modeling approach are discussed.

Storing information about experienced events in episodic memory requires the events’ constituent elements to be bound together. Such binding processes allow for a coherent retrieval of the experienced event. An event’s constituent elements may take very different forms such as persons, objects, locations, actions, and sensations. For example, imagine having bought bread at a bakery. Later remembering this particular event requires different elements such as the bakery (location), the bought bread (object), and the vendor (person) to be bound together in memory. If event elements are bound together, there should be an increased likelihood of retrieving subsequent events elements when a preceding element was successfully retrieved, thus leading to a stochastic dependency of the retrieval of event elements (e.g., Arnold et al., 2019; Boywitt & Meiser, 2012a, b; Horner et al., 2015; Horner & Burgess, 2013, 2014; Meiser and Bröder, 2002; Ngo et al., 2019; Starns & Hicks, 2005, 2008).

Much of the past research on binding in episodic memory (e.g., Balaban et al., 2019; Boywitt & Meiser, 2012a, b; Hicks and Starns, 2016; Meiser and Bröder, 2002; Starns & Hicks, 2005, 2008; Utochkin and Brady, 2020; Vogt and Bröder, 2007) investigated rather simple, item-based representations. Item-based representations consist of a single element with specific features, such as an object with a certain shape or color. Thus, item-based representations are static (see also Hunt & Einstein, 1981). More recently, research started to incorporate more complex, event-based representations that may include several elements (e.g., Andermane et al., 2021, Horner et al., 2015, 2013, 2014; James et al., 2020, Joensen et al., 2020). These elements may interact and thus, event-based representations are, at least potentially, dynamic (see also Rubin & Umanath, 2015). In this context, the presentation of different elements belonging to the same event may induce relational encoding with features common to the same event (Hunt & Einstein, 1981). Event-based representations can be considered to contain several item-based representations, with storage occuring in a hierarchical manner (i.e., item-based representations being nested in event-based representations, see Andermane et al., 2021) or event- and item-based representations can be distinguished based on different degrees of discrimination, with item-based representations containing more specific information than event-based representations (Hunt & Einstein, 1981). Additionally, event-based representations include a spatiotemporal context, which is not the case for item-based representations (e.g., Andermane et al., 2021). Contrary to item-based representations, event-based representations allow for the construction of scenes (Robin, 2018; Rubin & Umanath, 2015). This scene construction does not necessitate the exact remembering of the specific features of an event’s constituent elements (Rubin & Umanath, 2015). Most research on event-based representations has not considered specific features of the events’ constituent elements, which however have been a main focus of research on item-based representations (e.g., Balaban et al., 2019; Horner and Burgess, 2013; Joensen et al., 2020; Utochkin and Brady., 2020).

Because event-based representations are more complex than item-based representations, approaches for modeling stochastic dependencies of the retrieval of event elements developed for item-based representations can not be readily applied to event-based representations. Instead, different approaches have been proposed for event-based representations. The different approaches are first introduced before reporting a simulation study comparing the approaches regarding their power for detecting stochastic dependency of the retrieval of event elements and differences in dependency, Type I error rates, and susceptibility to variations in memory performance. The approaches are then applied to an empirical data example to evaluate the congruence of empirical inferences drawn by using the different approaches.

## Approach by Horner and Burgess

Horner and Burgess (2013) proposed a contingency-based approach that can be applied to data obtained from cued recognition or cued recall tasks. The approach considers items (i.e., test trials in a memory test) with a common cue or target as a dependency pair. For example, if events consist of the elements A, B, and C, the cue-target-pairs A–B and A–C may be considered a dependency pair. For each person *i*, event *t*, and dependency pair *jj’* a contingency table **X** showing the successful retrieval of the target of a dependency pair can be constructed, with 1 denoting successful retrieval and 0 a failure to retrieve the target:

1 $$ \textbf{X}_{\text{it}}^{\textrm{jj'}} = \left[ \begin{array}{rrr} j = 1, j' = 1 & j = 1, j' = 0 \\ j = 0, j' = 1 & j = 0, j' = 0 \end{array}\right] $$

Summing over events, a contingency table for a given person and dependency pair can be obtained:

2 $$ \textbf{X}_{\mathrm{i}}^{\textrm{jj'}} = \left[ \begin{array}{rrr} n_{11} & n_{10} \\ n_{01} & n_{00} \end{array}\right] $$

*n*_11_ is the frequency of both items of a dependency pair being correctly retrieved across events, *n*_10_ is the frequency of item *j* being correctly retrieved while item *j’* being incorrectly retrieved, *n*_01_ is the frequency of item *j* being incorrectly retrieved while item *j’* being correctly retrieved, and *n*_00_ is the frequency of both items being incorrectly retrieved. From these contingency tables (one per dependency pair), Horner and Burgess (2013) calculate a data-based measure of the dependency of the retrieval of event elements. The measure is first calculated for each dependency pair by summing the leading diagonal cells of each contingency table per person and dividing the results by the overall number of events *T*. Then the results are averaged across the set of dependency pairs *J*:

3 $$ D_{\textrm{HB, i}}^{\text{data}} = \frac{1}{|J|}\sum\limits_{jj' \in J}\frac{n_{11}+n_{00}}{T} $$

The measure reflects the mean proportion of items in an event that were both successfully or unsuccessfully retrieved. Because this measure necessarily increases if many (or few) event elements are successfully retrieved due to strong (or poor) overall memory performance, Horner and Burgess (2013) contrast it with dependency estimates from an “independent model,” which predicts a value of the measure under the assumption of independence based on the person’s mean performance for items of a dependency pair across events:

4 $$ \begin{array}{@{}rcl@{}} D_{\textrm{HB, i}}^{\text{ind}} &=& \frac{1}{|J|}\sum\limits_{jj' \in J}\left( \frac{n_{11}+n_{10}}{T}\frac{n_{11}+n_{01}}{T}\right.\\ &&\left.+\left( 1-\frac{n_{11}+n_{10}}{T}\right)\left( 1-\frac{n_{11}+n_{01}}{T}\right)\right) \end{array} $$

The actual dependency measure *D*_HB, i_ can then be obtained by subtracting *D* HB, iind from *D* HB, idata. The measure can take values between -1 and 1. A value of 0 indicates independence, positive values indicate dependency, and negative values indicate negative dependency such that the likelihood of retrieving an event element is smaller when a preceding event element was successfully retrieved.

## Yule’s Q

Similarly to the approach by Horner and Burgess (2013), one can calculate a measure of dependency from the contingency table in Eq. 2 using Yule’s Q (Yule, 1912; cf. Horner and Burgess, 2014; see also Hayman and Tulving, 1989; Kahana, 2002; Kahana et al., 2005), a commonly used measure of association in memory research. Yule’s Q is an odds ratio standardized to the value range of [-1, 1] with the same interpretation as the dependency measure by Horner and Burgess (2013). It is a special case of the gamma coefficient (Goodman & Kruskal, 1954) for 2 × 2 matrices and can be calculated as:

5 $$ Q_{\mathrm{i}}^{\textrm{jj'}} = \frac{n_{11}n_{00}-n_{10}n_{01}}{n_{11}n_{00}+n_{10}n_{01}} $$

As in the approach by Horner and Burgess (2013), one can then average across dependency pairs:

6 $$ Q_{\mathrm{i}} = \frac{1}{|J|}\sum\limits_{jj' \in J}Q_{\mathrm{i}}^{\textrm{jj'}} $$

## Adjusted Yule’s Q

A known problem of Yule’s Q is that zero frequencies cause it to become -1, 1, or undefined. One can circumvent this problem by adding a constant such as 0.5 to each cell of the contingency table in Eq. 2 (cf. Burton et al., 2019; Horner and Burgess, 2014). One can then calculate the adjusted Yule’s Q (*Q*_a_) as in Eqs. 5 and 6. However, as opposed to the approach by Horner and Burgess (2013), the approaches involving Yule’s Q do not attempt to correct for memory performance.

All the approaches mentioned so far are contingency-based, collapsing smaller contingency tables into a 2 × 2 contingency table per participant and dependency pair. Thus, the approaches may be prone to Simpson’s paradox (Hintzman, 1972, 1980; Simpson, 1951), meaning that if 2 × 2 contingency tables are collapsed into a summary one, the relationship of the two outcomes in the summary table may differ from the one shown in any of the original tables. This may occur due to confounding with participant differences, item differences, or participant-item interactions (Hintzman, 1972, 1980; see also Burton et al., 2017). Since the approaches compute participant-specific estimates, the problem of confounding with participant differences is avoided. However, potential confounding with item differences and, most notably, participant-item interactions remains an issue. Consequently, the approaches for estimating dependency using contingency analyses may be subject to problems of confounding.

## An IRT-based approach

Recently, Schreiner et al. (in press) proposed a measure of the retrieval of event elements based on item response theory (IRT, Lord, 1980; Lord and Novick, 1968). Contrary to the approaches outlined before, this measure is not contingency-based but operates on the level of individual item responses (i.e., test trial outcomes in a memory test). Thus, Simpson’s paradox does not apply. In addition, IRT jointly models participant differences, item differences, and participant-item interactions, thus avoiding confounding with these covariates. By using the three-parameter logistic model (Birnbaum, 1968), one can model the probability of person *i* to give a correct response *u* to item *j*, given a latent trait 𝜃, which represents memory performance in the current application of the model, an item difficulty β, an item-specific discrimination parameter α, and an item-specific guessing parameter γ:

7 $$ \textit{P}(\textit{u}_{\text{ij}} = 1) = {\upgamma}_{\mathrm{j}} + (1-{\upgamma}_{\mathrm{j}})\frac{e^{{\upalpha}_{\mathrm{j}}({\uptheta}_{\mathrm{i}}-{\upbeta}_{\mathrm{j}})}}{1+e^{{\upalpha}_{\mathrm{j}}({\uptheta}_{\mathrm{i}}-{\upbeta}_{\mathrm{j}})}} $$

In experimental settings, events are often randomly generated. Thus, it is often appropriate to fix the discrimination and guessing parameters. For example, when using cued recognition tests, it may be appropriate to fix the guessing parameter to the stochastic guessing probability derived from the number of response options (e.g., 0.2 for five response options). Discrimination parameters may be fixed to 1, as is the case in the Rasch model (Rasch, 1960), assuming all items having the same correlation or factor loading with the latent trait. When fixing the discrimination parameters to 1 and the guessing parameters to a constant *g*, the model is reduced to:

8 $$ \textit{P}(\textit{u}_{\text{ij}} = 1) = g + (1-g)\frac{e^{{\uptheta}_{\mathrm{i}}-{\upbeta}_{\mathrm{j}}}}{1+e^{{\uptheta}_{\mathrm{i}}-{\upbeta}_{\mathrm{j}}}} $$

This model assumes local independence (LI) of item responses, which means that all inter-item relationships are accounted for by the latent trait (de Ayala, 2009; Lazarsfeld and Henry, 1968). If the LI assumption holds, item residual correlations are zero. However, when binding of event elements occurs there are additional event-specific effects that violate the LI assumption. Consequently, item-residual correlations within events deviate from zero. Item-residual correlations can be estimated using the *Q*_3_ statistic (Yen, 1984), which is calculated for item pairs *jj’* in four steps: First, person and item parameters are estimated from the model in Eqs. 7 or 8. Second, the probability of correctly retrieving items *j* and *j’* is predicted from the model parameters. Third, the residuals for both items are calculated by subtracting the model-implied probability of a correct response from the observed response for each person. Finally, *Q*_3_ is calculated as the correlation of the residuals of both items. The *Q*_3_ statistic has an expected value of − 1*I*− 1 given LI, with *I* being the total number of items (Yen, 1993). Thus, *Q*_3_ is negatively biased and in an additional step a bias correction should be applied by subtracting the expected value from all *Q*_3_. Schreiner et al. (in press) then constructed a measure of the dependency of the retrieval of event elements ($D_{\mathrm {Q}_{3}}$) as the difference in mean within-event and mean between-event *Q*_3_:

9 $$ \textit{D}_{\mathrm{Q}_{3}} = \frac{1}{\textit{K}}\sum\limits_{{k>k^{\prime}}}\textit{Q}_{3}^{\text{kk}^{\prime}} - \frac{1}{\textit{L}}\sum\limits_{{l>l^{\prime}}}\textit{Q}_{3}^{\text{ll}^{\prime}} $$

where *kk’* are within-event item pairs, *ll’* are between-event item pairs, *K* is the total number of within-event item pairs and *L* is the total number of between-event item pairs. Given binding of event elements, within-event residual correlations deviate from zero and between-event residual correlations are close to zero. Consequently, $D_{\mathrm {Q}_{3}}$ deviates from zero. Like *D*_HB_ and Yule’s Q, $D_{\mathrm {Q}_{3}}$ can take values between -1 and 1 and its interpretation is equivalent to the former measures.

Because the sampling distribution of *Q*_3_, and consequently the one of $D_{\mathrm {Q}_{3}}$, is unknown (Chen and Thissen, 1997) and $D_{\mathrm {Q}_{3}}$ is an overall, not person-specific, measure of dependency, testing the dependency by means of *t*-tests or linear mixed models, which can be applied to the contingency-based approaches, is not possible. Instead, parametric bootstrapping can be applied, which is a simulation-based approach to generate data from estimated parameters to simulate a distribution of a statistic under the assumption that the data-generating model is true. There are generally two tests that are of interest: testing whether dependency is different from zero and testing whether dependency differs between experimental conditions or groups. For the first test, artificial response matrices can be repeatedly sampled from the model in Eq. 8, with item parameters and latent trait variance estimated from the original response matrix. For each simulated sample one can then calculate $D_{\mathrm {Q}_{3}}$ to obtain distributions under the null hypothesis of independence. From these distributions one can then calculate *p* values for the observed $D_{\mathrm {Q}_{3}}$. For the second test, the parametric bootstrap requires estimates of the event-specific effects, which can be obtained by fitting a bifactor model (see Gibbons and Hedeker, 1992; Wainer and Wang, 2000). This model extends the model in Eq. 7 by including additional event-specific latent traits λ:

10 $$ \textit{P}(\textit{u}_{\text{ij}} = 1) = {\upgamma}_{\mathrm{j}} + (1-{\upgamma}_{\mathrm{j}})\frac{e^{{\upalpha}_{\mathrm{j}}({\uptheta}_{\mathrm{i}}-{\upbeta}_{\mathrm{j}})-{\upalpha}_{\textrm{t(j)}}{\uplambda}_{\textrm{it(j)}}}}{1+e^{{\upalpha}_{\mathrm{j}}({\uptheta}_{\mathrm{i}}-{\upbeta}_{\mathrm{j}})-{\upalpha}_{\textrm{t(j)}}{\uplambda}_{\textrm{it(j)}}}} $$

with λ being the event-specific latent trait of person *i* for event *t(j)* to which item *j* belongs. When applying the same restrictions as in Eq. 8 the model reduces to:

11 $$ \textit{P}(\textit{u}_{\text{ij}} = 1) = g + (1-g)\frac{e^{{\uptheta}_{\mathrm{i}}-{\upbeta}_{\mathrm{j}}-{\uplambda}_{\textrm{it(j)}}}}{1+e^{{\uptheta}_{\mathrm{i}}-{\upbeta}_{\mathrm{j}}-{\uplambda}_{\textrm{it(j)}}}} $$

All latent traits in this model are mutually independent. The event-specific latent traits exert their influence via their variance. Higher variances indicate stronger event-specific effects. In experimental settings this model requires an additional latent trait for each event and thus quickly becomes very high-dimensional. It is thus advisable to put equality constraints on the event-specific trait variances within experimental conditions. Using the estimates of latent trait variances and item parameters one can then repeatedly sample artificial response matrices from the model in Eq. 11, while setting the latent trait variances equal to the ones of a given experimental condition (a reference condition). For example, when having two experimental conditions, one may set the latent trait variance of the second condition equal to the one of the first condition, making the model assume no difference in dependency between conditions. One can then calculate $D_{\mathrm {Q}_{3}}$ for each experimental condition and differences in $D_{\mathrm {Q}_{3}}$ between conditions to obtain distributions under the null hypothesis of equal dependency between conditions relative to the reference condition. From these distributions one can then calculate *p* values for the observed differences in $D_{\mathrm {Q}_{3}}$.

## Nonparametric variant of the IRT-based approach

While the previously presented IRT-based approach (Schreiner et al., in press) is parametric and requires the estimation of item and person parameters, Debelak and Koller (2020) recently proposed a nonparametric estimation procedure for *Q*_3_, building on the nonparametric testing framework by Ponocny (2001). Using a Markov-Chain Monte-Carlo algorithm by Verhelst (2008), a bootstrap sample of artifical response matrices with the same marginal sums as the original response matrix is generated. In the Rasch model (Rasch, 1960), and also the restricted model in Eq. 8, the marginal person sums are sufficient statistics for the general latent trait. It is then possible to estimate *P*(*u*_ij_ = 1) by averaging *u*_ij_ over all bootstrap samples. The nonparametric variant of *Q*_3_ is then computed like its parametric counterpart, using the estimated *P*(*u*_ij_ = 1) as the model-implied probability of a correct response. Based on the obtained nonparametric variants of *Q*_3_ one can then calculate a dependency measure ($D_{\mathrm {Q}_{3}}^{\text {np}}$) as in Eq. 9. Similarly as in the parametric approach it is then also possible to calculate $D_{\mathrm {Q}_{3}}^{\text {np}}$ for each bootstrap sample and to calculate *p* values for $D_{\mathrm {Q}_{3}}^{\text {np}}$ and differences in $D_{\mathrm {Q}_{3}}^{\text {np}}$.

Desirable properties for measures of binding effects in episodic memory are: high power in detecting stochastic dependency of the retrieval of event elements and differences in dependency, good maintenance of Type I error rates, and non-sensitivity to variations in memory performance. Type I error rates and power are central concepts for statistical hypothesis testing (see e.g., Cohen, 1988) in order to guarantee strict statistical tests and replicable findings. In addition, binding effects should be dissociated from memory performance, which requires measures of binding effects that are unaffected by memory performance, because otherwise it is unclear whether increased dependency of the retrieval of event elements can be attributed to actual binding effects or is due to higher levels of memory performance in the sample, which also increases the likelihood that several elements from the same event are correctly retrieved. In a simulation study we compared the five presented approaches regarding these criteria.

## Simulation study

### Methods

We conducted a Monte Carlo simulation. Responses were generated from the bifactor model in Eq. 11 with a global guessing parameter of *g* = 0.2, *t* = 30 events, and 6 items (i.e., test trials in a hypothetical memory test) per event, resulting in a total of *I* = 180 items. In an application, this scenario could be equivalent to testing each association of events consisting of three elements A, B, and C in both directions using a cued recognition task (i.e. testing the cue-target pairs A–B, B–A, A–C, C–A, B–C, and C–B). The different test trials represent the items. The simulation mimicked 2 experimental within-subjects conditions, resulting in 15 events and 90 items per experimental condition.

Item parameters were drawn from a standard normal distribution. Person parameters (i.e., latent memory proficiency [𝜃] and event-specific latent trait scores [λ_t_]) were drawn from a multivariate normal distribution with zero covariances, since the bifactor model assumes the general and event-specific latent traits to be mutually independent (e.g., Wang & Wilson, 2005). The mean of the general latent trait, representing overall memory performance, varied across simulation conditions the and variance was set to 5, based on empirical findings (cf. Schreiner et al., in press). The means of the event-specific latent traits were set to zero and the variances varied across simulation conditions. Variances were constrained to be equal within experimental conditions.

There were four design factors in the simulation: (a) sample size (*N* = {25, 50, 75, 100}), (b) dependency (event-specific trait variances, Dep. = {0, 0.5, 1}), (c) differences in dependency (differences in event-specific trait variances, Dep._diff_ = {0, 0.5, 1}), and (d) overall level of memory performance (mean of the general latent trait 𝜃, *P* = {-2, 0, 2}). Different levels of memory performance resulted in proportions of 40%-42% (*P* = -2), 59%-60% (*P* = 0), and 75%-80% (*P* = 2) correct responses. The sample sizes are normal to quite large for experimental studies of memory. The simulation conditions resulted from the fully crossed combination of the four design factors, resulting in 108 simulation conditions. For each of these, 1,000 response matrices were generated. For differences in dependency between conditions, the first experimental condition served as the reference condition. For the second experimental condition, the difference value was added to the dependency value of the first condition (i.e., the baseline dependency). Dependency values of zero indicate independence. For values larger zero there is positive dependency in the data. If the dependency difference is zero, the two experimental conditions are identical. Consequently, regarding results for testing against independence, only the results of the first experimental condition are reported. One limitation of $D_{\mathrm {Q}_{3}}$ is that the corresponding IRT model can not be estimated if there are items without variance because this prevents the estimation of item parameters for these items. To circumvent this problem in the simulation, the simulated data was redrawn until all items had non-zero variances.

The five dependency measures (*D*_HB_, *Q*, *Q*_a_^1^, $D_{\mathrm {Q}_{3}}$^2^, and $D_{\mathrm {Q}_{3}}^{\text {np}}$) were computed for each generated response matrix. Empirical detection rates were determined with the conventional significance level of α = 5% using one-tailed testing^3^ (dependency larger than zero for tests against independence and dependency lower in the first experimental condition than in the second experimental condition for tests of dependency differences). For *D*_HB_, *Q*, and *Q*_a_ one-sample *t*-tests against zero were conducted for tests against independence and paired *t*-tests were conducted for tests of dependency differences. For the parametric bootstrap required for $D_{\mathrm {Q}_{3}}$, the true parameter values (for fixed parameters) and correct distributional assumptions were used^4^. For each simulation condition, 1,000 bootstrap samples (cf. Davison & Hinkley, 1997) were generated prior to the simulation to obtain critical values for $D_{\mathrm {Q}_{3}}$. Note that item and person parameters were only drawn once per simulation condition for the parametric bootstrap. For $D_{\mathrm {Q}_{3}}^{\text {np}}$, 1,000 bootstrap samples were generated from each generated response matrix. These were used for the nonparametric estimation of *Q*_3_ (Debelak and Koller, 2020) and used to obtain critical values for $D_{\mathrm {Q}_{3}}^{\text {np}}$.

The simulation was conducted in the R Programming Environment (R Core Team, 2021) using the packages *SimDesign* (version 2.2, Chalmers & Adkins, 2020), *mirt* (version 1.33.2, Chalmers, 2012), and *eRm* (version 1.0-1, Mair et al., 2020; Mair and Hatzinger, 2007)^5^, and adapted functions from the package *sirt* (version 3.9-4, Robitzsch, 2020). Data and code for the simulation study are available via the Open Science Framework (OSF, https://osf.io/25mzu/).

### Results

**Fig. 1:**
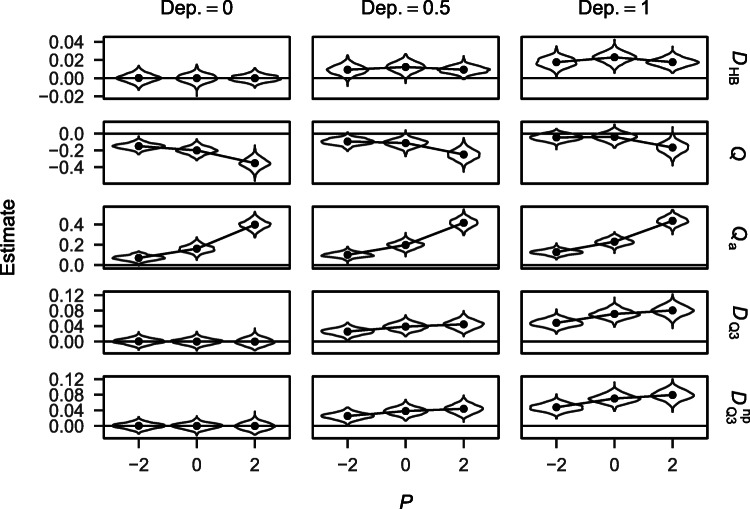
Dependency estimates and mean trajectories obtained from the different measures by dependency and performance for *N* = 100 . For *D*_HB_, *Q*, and *Q*_a_ the displayed values refer to the mean across participants within the different simulation conditions. Note the varying y scales for the different measures.

Figures referring to the distribution of dependency estimates (Figs. 1 and 4) show the values for a sample size of *N* = 100. Results for other sample sizes showed identical trends but distributions were more spread out due to larger standard errors. Because *D*_HB_, *Q*, and *Q*_a_ yield participant-specific estimates, the values shown in the figures refer to the respective means across participants. This applies for both types of tests (i.e., tests against independence and tests for differences in dependency between experimental conditions).

#### Testing Against Independence

##### Estimates

Figure 1 shows the distribution of dependency estimates yielded by the different approaches for the different simulation conditions. Given no dependency in the data, *D*_HB_, $D_{\mathrm {Q}_{3}}$, and $D_{\mathrm {Q}_{3}}^{\text {np}}$ were distributed around zero across performance conditions. *Q* on the other hand was negatively biased and *Q*_a_ was positively biased and both biases increased strongly with performance. All estimates increased with increasing dependency in the data. The sensitivity of *Q* and *Q*_a_ to performance was maintained if there was dependency in the data. In such cases, *D*_HB_, $D_{\mathrm {Q}_{3}}$, and $D_{\mathrm {Q}_{3}}^{\text {np}}$ also showed sensitivity to performance and this sensitivity increased with increasing dependency in the data, suggesting an interaction effect of dependency and performance on the estimates. *D*_HB_ showed the least sensitivity to performance and followed a curvilinear trend across performance conditions. $D_{\mathrm {Q}_{3}}$ and $D_{\mathrm {Q}_{3}}^{\text {np}}$ showed similar sensitivity to performance with a monotonic increase in estimates across performance conditions. Sensitivity to performance was higher than for *D*_HB_ but was still very small compared to *Q* and *Q*_a_.

In summary, *D*_HB_, $D_{\mathrm {Q}_{3}}$, and $D_{\mathrm {Q}_{3}}^{\text {np}}$ were robust against different degrees of overall performance given that there was no dependency in the data but were sensitive to performance if there was dependency in the data. This sensitivity increased with increasing dependency and was less pronounced for mean values of *D*_HB_. *Q* and *Q*_a_ were negatively and positively biased respectively and means were strongly affected by performance, even if there was no dependency in the data. Correlations between estimates of the different measures are shown in Table 1 in the Appendix.

##### Type I error rates

**Fig. 2:**
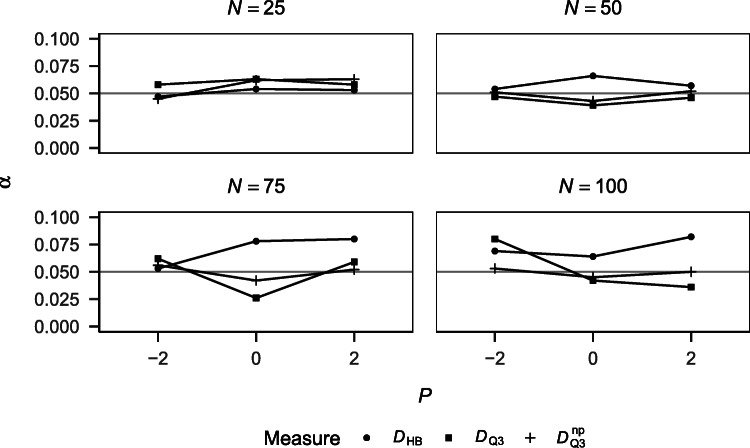
Type I error rates of the different measures for tests against independence by performance and sample size . *Q* and *Q*_a_ are not displayed.

Figure 2 shows the Type I error rates of the different approaches for the different simulation conditions. *Q*_a_ is not displayed because it yielded very high Type I error rates (> .41), which strongly increased with performance. This can be explained by its positive bias (see Fig. 1) and the one-tailed testing applied. *Q* is also not displayed because it yielded Type I error rates of zero in all conditions, which can be explained by its negative bias (see Fig. 1) and the one-tailed testing applied. *D*_HB_ tended to yield higher Type I error rates than $D_{\mathrm {Q}_{3}}$ and $D_{\mathrm {Q}_{3}}^{\text {np}}$ except for smaller sample sizes. There was no clear trend of Type I error rates across performance conditions, suggesting that the three measures yield Type I error rates that are unaffected by performance. $D_{\mathrm {Q}_{3}}$ and $D_{\mathrm {Q}_{3}}^{\text {np}}$ yielded Type I error rates close to 5%, suggesting good maintenance of the nominal significance level by these measures.

##### Power

**Fig. 3:**
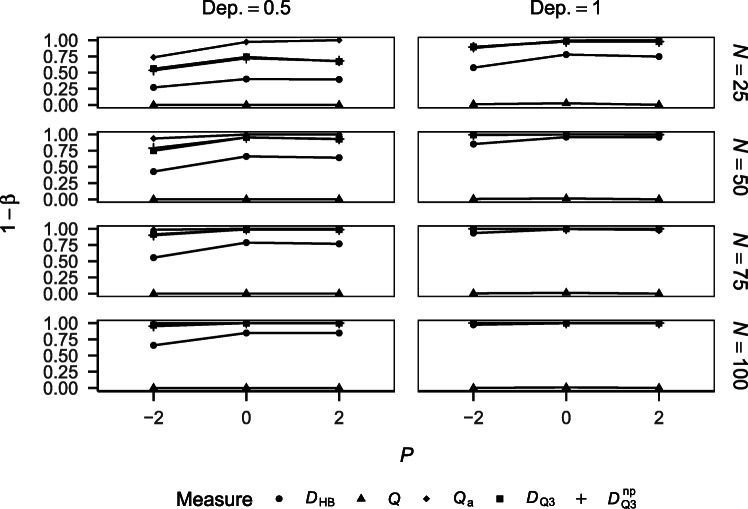
Power of the different measures for detecting dependency by performance, baseline dependency, and sample size

Figure 3 shows the power of the different approaches for detecting dependency for the different simulation conditions. Power increased with sample size and increasing dependency in the data. *Q* yielded very low power, which can again be explained by its negative bias (see Fig. 1). *Q*_a_ yielded very high power that is sensitive to performance. This can be explained by the measure’s positive bias (see Fig. 1). $D_{\mathrm {Q}_{3}}$ and $D_{\mathrm {Q}_{3}}^{\text {np}}$ yielded comparable power that was higher than the one yielded by *D*_HB_. The power yielded by all three measures was sensitive to performance but this sensitivity was comparable between the three measures.

#### Testing for differences in dependency

##### Estimates

**Fig. 4:**
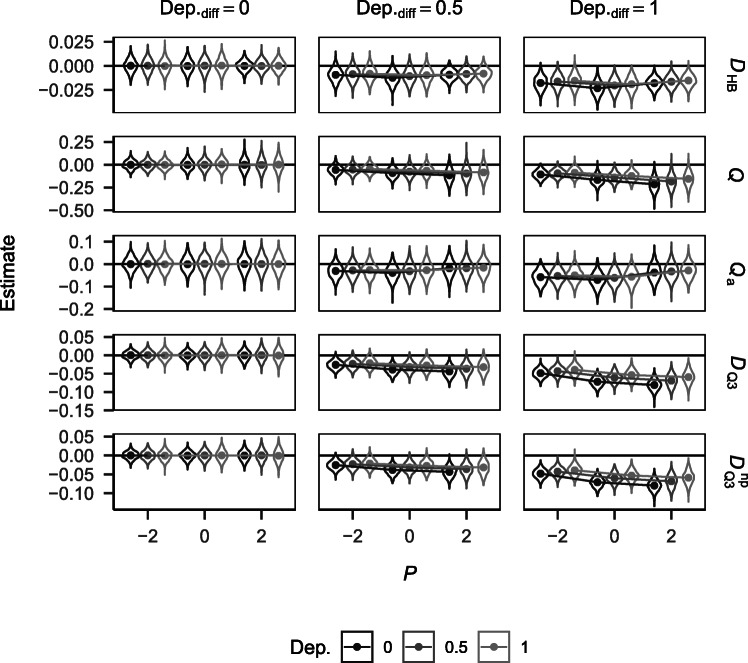
Estimates and mean trajectories of dependency differences obtained from the different measures by baseline dependency, dependency difference, and performance for *N* = 100 . For *D*_HB_, *Q*, and *Q*_a_ the displayed values refer to the mean differences across participants within the different simulation conditions. Note the varying y scales for the different measures.

Figure 4 shows the distribution of estimates of dependency differences yielded by the different approaches for the different simulation conditions. Given no difference between conditions, all estimates were distributed around zero, irrespective of performance and baseline dependency (i.e., dependency in the reference condition). All estimates decreased with increasing differences in dependency in the data. If there were dependency differences in the data, *D*_HB_ showed the least sensitivity to performance and followed a curvilinear trend across performance conditions. *Q* and *Q*_a_ were highly sensitive to performance. While *Q* monotonically increased with increasing performance, *Q*_a_ followed a curvilinear trend across performance conditions. $D_{\mathrm {Q}_{3}}$ and $D_{\mathrm {Q}_{3}}^{\text {np}}$ showed similar sensitivity to performance with a monotonic decrease in estimates across performance conditions. Sensitivity to performance was higher than for *D*_HB_ but was smaller than for *Q* and *Q*_a_. Sensitivity to memory performance increased with increasing differences in dependency for all measures. Finally, all estimates shifted closer to zero with an increasing baseline dependency. Correlations between estimates of dependency differences of the different measures are shown in Table 2 in the Appendix.

**Fig. 5:**
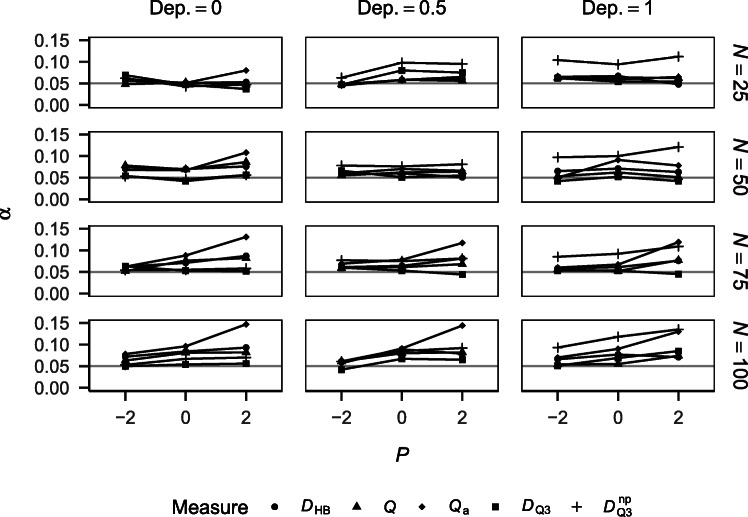
Type I error rates of the different measures for tests for differences in dependency by performance, baseline dependency, and sample size

##### Type I error rates

Figure 5 shows the Type I error rates of the different approaches when testing for differences in dependency for the different simulation conditions. *Q*_a_ and $D_{\mathrm {Q}_{3}}^{\text {np}}$ yielded the highest Type I error rates, whereas Type I error rates for *D*_HB_, *Q*, and $D_{\mathrm {Q}_{3}}$ were approximately comparable. Overall, $D_{\mathrm {Q}_{3}}$ showed the best maintenance of the nominal significance level. For $D_{\mathrm {Q}_{3}}^{\text {np}}$, Type I error rates increased with increasing baseline dependency. This was not the case for the other measures. There was no clear trend of Type I error rates across performance conditions, suggesting that the Type I error rates of the measures are unaffected by performance, except for *Q*_a_ for which Type I error rates increased with performance for larger sample sizes.

##### Power

**Fig. 6:**
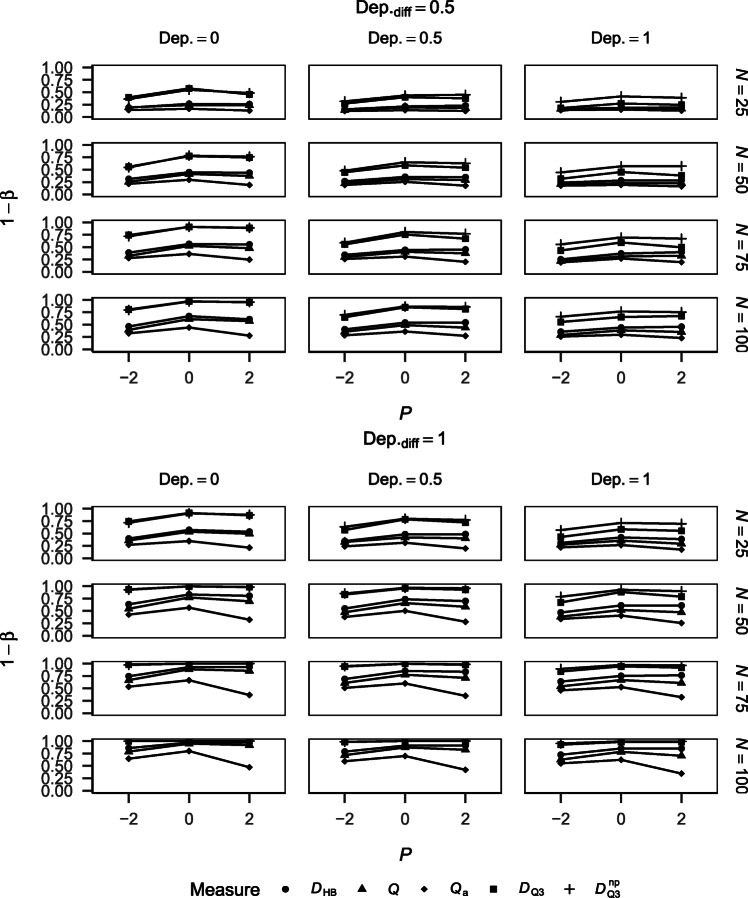
Power of the different measures for detecting differences in dependency by performance, baseline dependency, sample size, and dependency difference

Figure 6 shows the power of the different approaches for detecting differences in dependency for the different simulation conditions. Power increased with sample size and increasing dependency differences in the data and decreased with increasing baseline dependency for all measures. *Q*_a_ yielded the lowest power, followed by *Q*, and both measures were highly sensitive to performance, with a curvilinear trend across performance conditions. *D*_HB_ yielded higher power than *Q*_a_ and *Q* but lower power than $D_{\mathrm {Q}_{3}}$ and $D_{\mathrm {Q}_{3}}^{\text {np}}$. *D*_HB_ was sensitive to performance, either monotonically increasing with performance or showing a curvilinear trend across performance conditions, but the sensitivity to performance was lower than for *Q*_a_ and *Q*. $D_{\mathrm {Q}_{3}}$ and $D_{\mathrm {Q}_{3}}^{\text {np}}$ yielded the highest power, with slightly higher power for $D_{\mathrm {Q}_{3}}^{\text {np}}$ than $D_{\mathrm {Q}_{3}}$. This difference increased with increasing baseline dependency and may be explained by the increased sensitivity of $D_{\mathrm {Q}_{3}}^{\text {np}}$ given a higher level of dependency in the data, which also manifested in higher Type I error rates (see Fig. 5). $D_{\mathrm {Q}_{3}}$ and $D_{\mathrm {Q}_{3}}^{\text {np}}$ were similarly sensitive to performance as *D*_HB_, either monotonically increasing with performance or showing a curvilinear trend across performance conditions.

### Discussion

The simulation showed that *Q* yields negatively biased and *Q*_a_ yields positively biased estimates, even if there is no dependency in the data. This also manifests in very high Type I error rates for *Q*_a_ and very low power for detecting stochastic dependency of the retrieval of event elements for *Q*. The measures perform somewhat better when testing for differences in dependency between experimental conditions but are still inferior to the other measures. The two measures are also strongly affected by varying levels of overall memory performance, since they do not attempt to correct for memory performance as do *D*_HB_, $D_{\mathrm {Q}_{3}}$, and $D_{\mathrm {Q}_{3}}^{\text {np}}$. The latter three measures yield unbiased estimates and are unaffected by varying levels of overall memory performance given no dependency or no difference in dependency. However, if there is dependency or there are differences in dependency, all three measures are affected by memory performance, although to a much smaller extent than *Q* and *Q*_a_. In such cases, the power of *D*_HB_, $D_{\mathrm {Q}_{3}}$, and $D_{\mathrm {Q}_{3}}^{\text {np}}$ is affected to a similar degree, even though the mean estimates of *D*_HB_ across participants are least affected by memory performance. Note however, that person-specific estimates may be more strongly affected by memory performance. *D*_HB_ is affected by memory performance because the data-based dependency estimate and the dependency estimate from the independent model do not scale perfectly equal with memory performance. For $D_{\mathrm {Q}_{3}}$ this may be because fitting a unidimensional IRT model to locally dependent data leads to overestimation of measurement precision (Ip, 2010; Wainer and Wang, 2000) and worse recovery of person parameters (Koziol, 2016). Similar problems may arise for $D_{\mathrm {Q}_{3}}^{\text {np}}$. While it does not require the estimation of person parameters, it builds on the property of sum scores as sufficient statistics in the Rasch model (Rasch, 1960), which assumes local independence. $D_{\mathrm {Q}_{3}}$ and $D_{\mathrm {Q}_{3}}^{\text {np}}$ yield higher power than *D*_HB_, emphasizing the advantage of running analyses on individual item responses rather than aggregated contingency tables. However, when testing for differences in dependency, $D_{\mathrm {Q}_{3}}^{\text {np}}$ yields increased Type I error rates with increasing dependency in the data. Since *D*_HB_ and $D_{\mathrm {Q}_{3}}$ are unbiased under the null hypothesis and their Type I error rates are unaffected by memory performance and baseline dependency (for $D_{\mathrm {Q}_{3}}^{\text {np}}$ this holds for single parameter tests, but not for tests of parameter differences), their susceptibility to memory performance reduces to a power problem when focusing on statistical inferences rather than descriptive estimates.

Overall, $D_{\mathrm {Q}_{3}}$ performed best because it yields unbiased estimates under the null hypothesis, provides good maintenance of Type I error rates that tend to be better than that of *D*_HB_ and $D_{\mathrm {Q}_{3}}^{\text {np}}$, especially when testing for differences in dependency, and yields high power, although power is similarly affected by memory performance as is the power of *D*_HB_ and $D_{\mathrm {Q}_{3}}^{\text {np}}$. Next, we applied the different measures to an empirical example to compare the congruence of inferences drawn from empirical data.

## Empirical application

### Methods

As an empirical data example, a dataset by James et al. (2020, Experiment 1), was used (the original data is available at https://osf.io/cqm7v/). In this experiment 45 participants were presented events consisting of an animal, an object, and a location. Event elements were presented as cartoon illustrations, which were additionally named aloud through headphones. There were 2 experimental conditions, which were administered in a within-subjects design and with 15 events presented in each condition. In the simultaneous encoding condition all event elements were presented together in a single learning trial. In the separated encoding condition each pairwise association between event elements was presented separately across three learning trials. After encoding, participants conducted a cued recognition test with four response alternatives and six test trials per event (all associations were tested in both directions), resulting in 180 items. Mean memory performance was .71 in the simultaneous encoding condition and .73 in the separated encoding condition, making the setting similar to the simulation conditions with *P* = 2. Previous studies found a significant positive dependency in both a simultaneous and a separated encoding condition, with no significant difference in dependency between conditions (Bisby et al., 2018; Horner and Burgess, 2014).

The five dependency measures were computed based on the data, using a significance level of α = 5% (two-tailed testing). For computing $D_{\mathrm {Q}_{3}}$, *g* was set to the stochastic guessing probability of 0.25 given four response alternatives^6^. The analysis scripts for the empirical application are available via the OSF (https://osf.io/25mzu/).

### Results

**Fig. 7:**
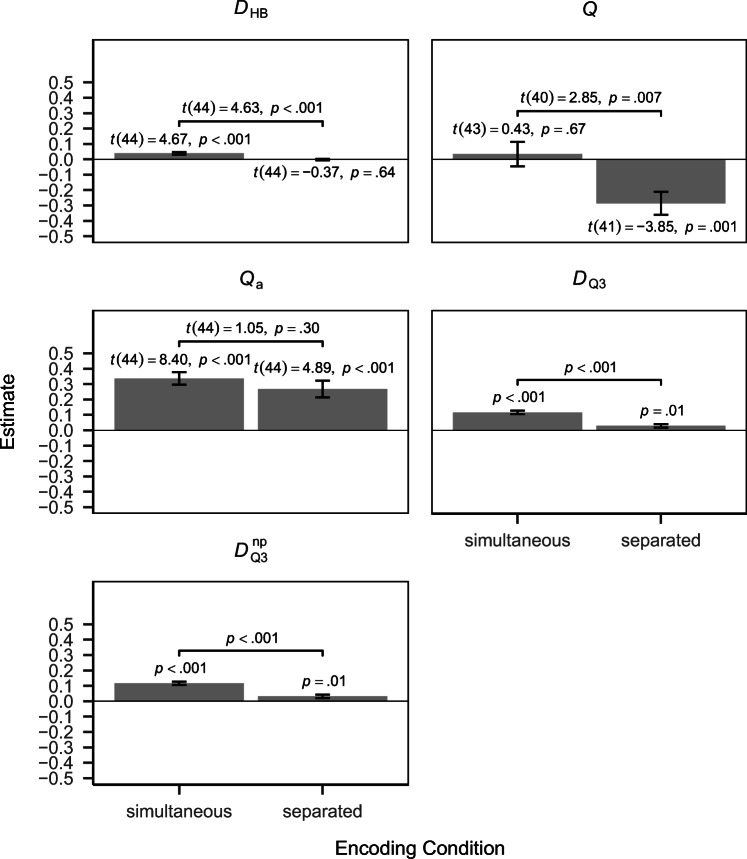
Results for the data of Experiment 1 by James et al., (2020) using the different approaches

Results using the different dependency measures are shown in Fig. 7. The results for *D*_HB_ are in accordance with those reported by James et al., (2020) — there was a significant positive dependency in the simultaneous encoding condition but not in the separated encoding condition, with a significant difference between conditions. This contradicts previous findings by Horner and Burgess (2014) and Bisby et al., (2018), which found a significant positive dependency also in the separated encoding condition and no difference in dependency between conditions. $D_{\mathrm {Q}_{3}}$ and $D_{\mathrm {Q}_{3}}^{\text {np}}$ yielded similar results as *D*_HB_. However, using these measures the dependency in the separated encoding condition was also positive and significant, with the difference between conditions still being significant. Since $D_{\mathrm {Q}_{3}}$ and $D_{\mathrm {Q}_{3}}^{\text {np}}$ yield higher power for detecting dependencies than *D*_HB_ it may be the case that the power of *D*_HB_ is insufficient for detecting the weak dependency in the separated encoding condition. The results using $D_{\mathrm {Q}_{3}}$ and $D_{\mathrm {Q}_{3}}^{\text {np}}$ are also more consistent with the findings by Horner and Burgess (2014) and Bisby et al., (2018) in the sense that they also found a positive dependency in the separated encoding condition. However, they are consistent with the finding by James et al., (2020) that there is a significant difference in dependency between conditions, which was not found by Horner and Burgess (2014) and Bisby et al., (2018).

*Q* and *Q*_a_ yielded very different results than the other measures. Using *Q*, there was no significant dependency in the simultaneous encoding condition and a significantly negative dependency in the separated encoding condition, with a significant difference between conditions. Using *Q*_a_ there was a significant positive dependency in both conditions but the difference between conditions was non-significant. These divergent findings may be explained by the negative bias of *Q* and the positive bias of *Q*_a_. The results using *Q* are quite inconsistent with previous findings and are only partially consistent with the findings by James et al., (2020) in the sense that there is a significant difference in dependency between conditions. While the results using *Q*_a_ are actually in accordance with the findings by Horner and Burgess (2014) and Bisby et al., (2018), the results from the simulation study and the incongruence with results using the other measures indicate that this result is likely not a correct representation of the given data.

## General discussion

In the current research we compared five approaches for measuring binding effects (i.e., stochastic dependencies of the retrieval of event elements) in event-based episodic representations regarding their empirical detection rates, susceptibility to memory performance, and congruence of empirical estimates. The approaches based on Yule’s Q (*Q* and *Q*_a_, Yule, 1912; cf. Horner & Burgess, 2014) yield biased estimates, with *Q* being negatively and *Q*_a_ being positively biased. In addition, the measures are highly susceptible to memory performance and applying them to the empirical example lead to considerable deviations from the results obtained by applying the other approaches. Thus, *Q* and *Q*_a_ are unsuitable for measuring binding effects in event-based episodic representations. The approach by Horner and Burgess (2013, *D*_HB_), the IRT-based approach ($D_{\mathrm {Q}_{3}}$, Schreiner et al., in press), and the nonparametric variant of the IRT-based approach ($D_{\mathrm {Q}_{3}}^{\text {np}}$, cf. Debelak and Koller, 2020; Schreiner et al., in press) are unbiased and not susceptible to memory performance under the null hypothesis of no dependency in the data or no differences in dependency between conditions. They are however affected by performance if there is dependency in the data or there are differences in dependency between conditions. This affects the power of all three measures to a similar degree. Since memory performance affects the power but not the Type I error rates of these measures, they do not elicit artifactual binding results as a consequence of base performance. This is because, when focusing on statistical inferences, the sensitivity of the measures is only affected if there is a true binding effect, reducing the effect of memory performance to a power problem. $D_{\mathrm {Q}_{3}}$ and $D_{\mathrm {Q}_{3}}^{\text {np}}$ yield higher power than *D*_HB_. However, $D_{\mathrm {Q}_{3}}^{\text {np}}$ yields increased Type I error rates with increasing dependency in the data when testing for differences in dependency between conditions. Compared to $D_{\mathrm {Q}_{3}}$, $D_{\mathrm {Q}_{3}}^{\text {np}}$ yielded, on average, Type I error rates increased by 0.003 if the baseline dependency was 0, 0.02 if the baseline dependency was 0.5, and 0.05 if the baseline dependency was 1. Applying *D*_HB_, $D_{\mathrm {Q}_{3}}$, and $D_{\mathrm {Q}_{3}}^{\text {np}}$ to the empirical example lead to similar results, but the results obtained by applying $D_{\mathrm {Q}_{3}}$ and $D_{\mathrm {Q}_{3}}^{\text {np}}$ were more consistent with previous findings by Horner and Burgess (2014) and Bisby et al., (2018). Given that memory performance in the empirical example was relatively high and similar to the simulation conditions with *P* = 2, the estimates for $D_{\mathrm {Q}_{3}}$ and $D_{\mathrm {Q}_{3}}^{\text {np}}$ may be somewhat inflated, given that estimates for these measures tend to increase with performance, and more so than the mean values of *D*_HB_. However, Type I error rates of the two measures do not increase with performance. Thus, the statistical inference that there is a significant positive dependency in the separated encoding condition can not be attributed to inflated sensitivity of $D_{\mathrm {Q}_{3}}$ and $D_{\mathrm {Q}_{3}}^{\text {np}}$ due to high memory performance. Taking together the simulation results and the results from the empirical application, $D_{\mathrm {Q}_{3}}$ performed best among the five measures. It provides unbiased estimates under the null hypothesis, provides good maintenance of Type I error rates that are unaffected by memory performance and baseline dependency, yields high power (subject to memory performance like *D*_HB_ and $D_{\mathrm {Q}_{3}}^{\text {np}}$), and yielded results for the empirical example that are more consistent with findings of previous studies (Bisby et al., 2018; Horner and Burgess, 2014).

A potential limitation concerning the results may be that both $D_{\mathrm {Q}_{3}}$ and the data generation procedure were IRT-based and we used the true discrimination parameters and distributional assumptions for computing $D_{\mathrm {Q}_{3}}$. This may have provided $D_{\mathrm {Q}_{3}}$ with some advantage over the other approaches. However, we chose the data generation procedure because it reflects well the actual psychological processes in memory retrieval given binding effects. In that sense, one could argue that $D_{\mathrm {Q}_{3}}$ is a better approximation of the psychological processes that underlie binding effects than are the other approaches. Further, $D_{\mathrm {Q}_{3}}$ should be rather robust against misspecifications of certain model parameters or distributional assumptions, since such misspecifications affect both within- and between-event residual correlations, which are contrasted in the computation of $D_{\mathrm {Q}_{3}}$. The finding that misspecification of the guessing parameter in the simulation study did not substantially affect the results supports this notion. Nevertheless, the robustness of $D_{\mathrm {Q}_{3}}$ against misspecifications of model parameters and distributional assumptions should be examined in future research.

$D_{\mathrm {Q}_{3}}$ provides some additional advantages. First, it operates on the level of individual item responses rather than aggregate contingency tables as do *D*_HB_, *Q*, and *Q*_a_, and the IRT model on which the measure is based considers participant and item differences as well as participant-item interactions. Thus, contrary to the contingency-based approaches, $D_{\mathrm {Q}_{3}}$ is not prone to Simpson’s paradox (Hintzman, 1972, 1980; Simpson, 1951; see also Burton et al., 2017). Second, IRT-based measures enable established and plausible modeling of meaningful psychological variables instead of running analyses on the basis of descriptive contingency tables. Third, $D_{\mathrm {Q}_{3}}$ can in principle be applied to a greater variety of testing procedures than the contingency-based approaches. The contingency-based approaches require some common feature of items for identifying the dependency pairs, such as items having a common cue or target element. If testing situations do not involve cueing, such identifying features are absent and consequently, dependency pairs would be arbitrary. Since $D_{\mathrm {Q}_{3}}$ does not require such identifying features (the assignment of items to a common event is sufficient), it can in principle also be applied to testing situations not involving cueing such as free recall or free recognition. For example, imagine participants are presented three words in a joint temporal context at a time, forming an event. Then, each word can form a binary item that is assigned the value 1, if the word has been successfully recalled or recognized, and 0 if not, resulting in three items per event. One can then compute $D_{\mathrm {Q}_{3}}$ the same way as for cued recognition output, based on the residual correlations between item pairs. Yet, evaluating the consistency of $D_{\mathrm {Q}_{3}}$, and also the other approaches, across different types of memory tests is an interesting prospect for future research. This would likely require a systematic investigation of several empirical data sets, which used various types of memory tests, or conducting an experiment with a given paradigm and varying the type of memory test between participants. Fourth, $D_{\mathrm {Q}_{3}}$ can in principle be extended to account for polytomous instead of dichotomous item responses, for example by using the rating scale model (Andrich, 1978) or the partical credit model (Masters, 1982) as the basis for computing the *Q*_3_ statistics. Finally, the approach yields estimated person and item parameters as useful by-products of the dependency analysis. For example, in applications with fixed event composition rather than random assignment of elements to events, item parameters may be used to identify problematic events with, for example, very high or very low difficulty of the associated items to improve the study material for subsequent experiments. Person parameters may be used to compare participants regarding their overall memory performance (but note that estimation of person parameter may be negatively affected by binding effects resulting in locally dependent data, see Koziol, 2016). However, some further considerations have to be taken into account when selecting a suitable measure for a given setting.

First, $D_{\mathrm {Q}_{3}}$ yields an overall or condition-specific dependency estimate. In some cases it may be necessary to obtain person-specific dependency estimates, which are not provided by $D_{\mathrm {Q}_{3}}$ in its current implementation. These are however provided by *D*_HB_ and one may use this measure in such cases. Second, if one wants to use $D_{\mathrm {Q}_{3}}$ and there are items without variance, item parameters for these items can not be estimated. In such a case one would have to exclude these items or reorder items if possible. The risk of this to occur increases with smaller sample sizes, increasing prevalence of missing values, and more extreme levels of memory performance. In the simulation, this issue was actively prevented by resampling until there were no items without variance. Still, there were some convergence issues for small sample sizes. Third, the bootstrap approach for $D_{\mathrm {Q}_{3}}$ is currently only designed for the comparison of two conditions, thus only enabling pairwise comparisons when using $D_{\mathrm {Q}_{3}}$. Finally, power is not the only issue to consider when determining sample size when using $D_{\mathrm {Q}_{3}}$. Parameter estimation becomes more stable with increasing sample size. This leads to more reliable estimates and may enable one to freely estimate parameters that may have to be fixed for smaller sample sizes, for example discrimination or guessing parameters, making the measure more flexible. In summary, we recommend to use $D_{\mathrm {Q}_{3}}$ as a measure of binding effects in event-based episodic representations if the mentioned considerations have been taken into account.

## Data Availability

The data and code are available via the Open Science Framework (https://osf.io/25mzu/). The study was not preregistered.

## Appendix: Correlations Between Dependency Estimates

**Table 1:** Mean [Range] of Correlations Between Dependency Estimates of the Different Measures Across Simulation Conditions

	*D*_HB_	*Q*	*Q*_a_	$D_{\mathrm {Q}_{\textrm {3}}}$
*D*_HB_	1			
*Q*	.82 [.54, .94]	1		
*Q*_a_	.65 [.13, .93]	.41 [-.19, .85]	1	
$D_{\mathrm {Q}_{\textrm {3}}}$	.78 [.44, .94]	.64 [.23, .89]	.50 [.12, .83]	1
$D_{\mathrm {Q}_{\textrm {3}}}^{\text {np}}$	.79 [.44, .94]	.64 [.24, .89]	.50 [.13, .84]	.99 [.96, >.99]

*Notes*. For *D*_HB_, *Q*, and *Q*_a_ correlations refer to the mean values of the respective estimates. For computing the mean correlations, Fisher’s Z-transformation was applied.

**Table 2:** Mean [Range] of Correlations Between Estimates of Dependency Differences of the Different Measures Across Simulation Conditions

	*D*_HB_	*Q*	*Q*_a_	$D_{\mathrm {Q}_{\textrm {3}}}$
*D*_HB_	1			
*Q*	.78 [.65, .85]	1		
*Q*_a_	.85 [.60, .93]	.64 [.19, .83]	1	
$D_{\mathrm {Q}_{\textrm {3}}}$	.60 [.41, .70]	.44 [.31, .58]	.50 [.25, .66]	1
$D_{\mathrm {Q}_{\textrm {3}}}^{\text {np}}$	.61 [.44, .71]	.45 [.32, .58]	.51 [.27, .67]	.98 [.96, .99]

*Notes*. For *D*_HB_, *Q*, and *Q*_a_ correlations refer to the mean values of the respective difference estimates. For computing the mean correlations, Fisher’s Z-transformation was applied.

## Footnotes

1 *Q*_a_ was computed by adding the constant 0.5 to each contingency table.

2 While it may conceptually often make sense to set the guessing parameter to the stochastic guessing probability given some number of response alternatives, the true guessing parameters in the sample may deviate from this probability, for example due to participants using strategies that increase their probability of a correct response. Thus, we computed $D_{\mathrm {Q}_{3}}$ with different degrees of misspecification of the guessing parameter — *g* = 0.2 (no misspecification), *g* = 0.15 (underestimation), and *g* = 0.25 (overestimation). Over- or underestimation of the guessing parameter did not substantially affect the results and only the results with no misspecification of the guessing parameter are reported.

3 We used one-tailed testing because the data generation process does not allow for negative dependencies (variances of the event-specific latent traits can not be negative).

4 In practice one would have to estimate item parameters and latent trait variances from the data by initially fitting a unidimensional model (for tests against independence) or a bifactor model (for tests for differences between conditions).

5 The *eRm* package was used for computing $D_{\mathrm {Q}_{3}}^{\text {np}}$. To do this, some of the package functions needed to be adjusted. The adjusted functions are available via the OSF.

6 Given that associations were tested in both directions, it may be possible that guessing differed between the first and second test of an association within an event. However, in the absence of more specific information and also considering model parsimony, we considered the stochastic guessing probability to be the most objective and appropriate criterion.
